# Temporal contrast adaptation in the analysis of visual function in primary open-angle glaucoma

**DOI:** 10.1007/s00417-022-05619-4

**Published:** 2022-03-16

**Authors:** Qianru Wu, Minyue Xie, Xuhao Chen, Di Zhang, Xiaoyong Chen, Ke Xu, Ying Hong, Chun Zhang

**Affiliations:** 1 Department of Ophthalmology, Beijing Key Laboratory of Restoration of Damaged Ocular Nerve, Peking University Third Hospital, 49 North Garden Road, Haidian District, Beijing, 100191 China; 2 Beijing Tongren Eye Center, Beijing Tongren Hospital, Beijing Institute of Ophthalmology, Capital Medical University, Beijing, 100730 China

**Keywords:** Primary open-angle glaucoma, Visual function analysis, Temporal contrast adaptation

## Abstract

**Purpose:**

To explore the utility of the recovery time (RT) after temporal contrast adaptation in primary open-angle glaucoma (POAG) visual function analysis, especially in severe and end-stage glaucoma, by the Erlanger Flicker Test (EFT).

**Methods:**

This study included 80 POAG eyes (45 subjects) and 20 normal eyes (20 subjects). POAG eyes were divided into 5 groups. The diagnostic efficacy of the EFT was assessed, and the RT of POAG eyes at different stages was compared. The EFT results were compared with glaucomatous structure and function test results. A nomogram was developed to predict disease progression by the RT and structural indicators.

**Results:**

In the normal eyes, as the test contrast increased, the RT gradually decreased. The EFT test–retest reproducibility was good, with intraclass correlation coefficient values of 0.6 (*P* < 0.05) for each test contrast. At 12%, 25%, and 35% contrast, the RT in the severe and end-stage glaucoma eyes was significantly prolonged compared with the control group (*P* < 0.05). The RT at different contrasts was significantly correlated with visual acuity, mean defect, mean sensitivity, and general and individual quadrant optic nerve fiber layer thickness (*P* ≤ 0.001). The receiver operating curve indicated that RT_12%_ showed the best overall area under the curve (0.863). We included RT_25%_ and average optic nerve fiber layer thickness in constructing the nomogram. POAG eyes were further divided into 8 stages. According to the probability distribution, this model showed good performance for visual function analysis in advanced glaucoma.

**Conclusions:**

Combined with traditional glaucomatous structural and functional parameters, the EFT can be used in the diagnosis and visual function analysis of POAG, especially for severe and end-stage glaucoma. It could be a potential test for disease staging in severe and end-stage glaucoma.

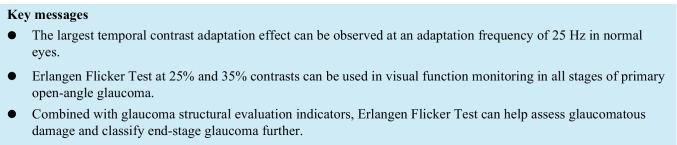

## Introduction

Glaucoma is the leading cause of irreversible blindness worldwide, and the number of people with glaucoma is expected to increase to 111.8 million by 2040 [1]. Primary open-angle glaucoma (POAG) is the most common type of glaucoma [2]. Due to progressive visual field defects, most patients with POAG will have substantial visual dysfunction and a significantly decreased vision-related quality of life [3].

At present, modern technologies such as standard achromatic perimetry (SAP) and optical coherence tomography (OCT) are valuable tools for monitoring the structural and functional changes in glaucoma patients [4]. However, for patients with advanced or end-stage glaucoma, the effectiveness of these technologies is limited. As the disease progresses, the parameters of OCT, such as the retinal nerve fiber layer thickness (RNFLT), ganglion cell inner plexiform layer, or ganglion cell complex, will be subjected to a floor effect [5–9]. The worse the glaucomatous damage is, the lower the accuracy of these parameters may be [10], which makes them unable to detect glaucomatous progression. The diagnostic value of SAP is also limited for advanced glaucoma, although visual field tests are an important method for the clinical detection of glaucomatous progression. Due to the severe visual field loss in patients with advanced glaucoma, only tubular vision remains, and fixation loss is common, therefore increasing the false-positive or false-negative rate of the visual field test. In some cases of end-stage glaucoma, when it exceeds 20 dB, the mean deviation (MD) remains unchanged during the follow-up period, and some methods for visual field progression detection, such as guided progression analysis or pointwise linear regression, have a lower accuracy as the visual field deteriorates and the follow-up time increases [11].

Recent studies have shown that glaucoma is not only an ophthalmic disease, but also a complex lesion involving the entire visual pathway, including ganglion cells, the optic nerve, ophthalmic tract, lateral geniculate body, and visual center [12]. The loss of retinal ganglion cells (RGCs) has been considered the primary pathogenesis of glaucoma [13]. Specific subtypes of RGCs, such as magnocellular (M) cells and parvocellular (P) cells, transfer visual information through the dorsal lateral geniculate nucleus (LGN) and finally to the visual cortex. Studies have shown that among all types of RGCs, M cells may be selectively damaged in glaucoma, resulting in visual field loss [14–16].

Studies have shown that electrophysiological tests, such as electroretinogram or visual evoked potential, have a certain value for the diagnosis and disease monitoring in glaucoma [17–19]. However, due to strict testing requirements, such as darkrooms and quiet environments, there are several limitations for electrophysiological tests in the detection of glaucomatous progression. The Erlangen Flicker Test (EFT) is a psychophysical method that uses temporal contrast sensitivity to monitor glaucomatous dysfunction by quantifying M-cell responses [20]. Studies have shown that temporal contrast adaptation originates from the retina or cortex and that temporal contrast adaptation of cortical origin occurs at temporal frequencies below 4 Hz [21]. At higher temporal frequencies, only the magnocellular pathway produces contrast adaptation [22]. Hohberger et al. [20] showed that in healthy people, the largest adaptation effect was observed at an adaptation frequency of 25 Hz. Due to the self-protection mechanism of optic nerve cells, after high-contrast stimulation, low-contrast stimulation can be perceived only after a certain time. The interval in which the lower-contrast can be perceived between two consecutive stimulations is defined as the recovery time (RT), that is, the time interval in which the ganglion cells can sense the next stimulation, which can be used to reflect glaucomatous optic nerve damage [23]. The purpose of this study is to investigate the efficacy of the EFT in detecting the visual function of POAG patients and its ability to further classify glaucoma, especially in advanced or end-stage glaucoma patients who have severe visual field defects.

## Materials and methods

### Subjects

This prospective study was performed in the Department of Ophthalmology, Peking University Third Hospital, from January 2020 to January 2021. The study was performed in accordance with the tenets of the Declaration of Helsinki. All participants provided informed consent, which was approved by the Peking University Third Hospital Medical Science Research Ethics Committee. Forty-five POAG patients (80 eyes) and twenty normal controls (20 eyes) were included in this study. Only one eye of each normal subject was enrolled in the study and it was randomly selected. All patients underwent complete ophthalmic examination, including best corrected visual acuity (BCVA), intraocular pressure (IOP) measurement, fundoscopy (Carl Zeiss Meditec, Jena, Germany), OCT scanning (Heidelberg Engineering, Heidelberg, Germany), and visual field test performed by Octopus perimetry (Octopus 101, Haag-Streit AG, Switzerland). Normal eyes were those with a normal IOP and no history of IOP elevation, normal optic disks, no optic nerve damage, and normal visual field test results. Additionally, these eyes had no history of ocular disease or previous ophthalmic surgery. POAG was diagnosed according to the guidelines of the American Academy of Ophthalmology [24], including open anterior chamber angles observed with gonioscopy with or without an elevated IOP, glaucomatous optic nerve damage detected by optic disc and RNFL examination, such as thinning of the RNFL and notching of the neuroretinal rim, and visual field defects on SAP. General exclusion criteria were significant lens or corneal opacities, retinopathy, optic nerve damage, or visual field defects caused by trauma, uveitis, exfoliation syndrome, or other systemic diseases, other ocular diseases that can cause secondary glaucoma or a previous history of intraocular surgery, or mental illness, such as dementia and Parkinson.

### Erlanger Flicker Test

The Erlanger Flicker Test was performed by a full-field stimulator (Ganzfeld bowl Q450F, Roland Consult, Germany) equipped with white light-emitting diodes (LEDs) that provided the stimulation. The light intensity changed in a sinusoidal manner and was spatially homogeneous. The contrast and the frequency of the stimuli could be set variably [25]. The adaptation frequency was 25 Hz, and the average luminance was 49.5 cd/m^2^ [23]. The test was performed with one eye at a time, with the contralateral eye covered by an eye patch. The stimulus was divided into two parts: the adaptation stimulus and the test stimulus. After a full-field adaptation stimulus (100% contrast), a test stimulus with a lower contrast was presented. The test stimulus was presented at 3%, 5%, 12%, 25%, and 35% contrast for normal eyes and 12%, 25%, and 35% contrast for POAG eyes. After perceiving the adaptation stimulus, the patient might not immediately perceive the test stimulus. When the patient ultimately perceived the lower contrast test stimulus, he or she was asked to press the button in his or her hand, and the system automatically recorded the time from the end of the adaptation stimulus to the perception of the test stimulus (RT). The current study adopted 15 s as the duration of the adaptation stimulus, and the perception period was 50 s. When the patient pressed the button, the system jumped to the next test. The whole trial was repeated 4 times. The first trial was not included in the measurement; the mean RT was the average of the RTs of the last three trials, which was recorded as the result for the EFT. The RTs at 3% contrast (RT_3%_), 5% contrast (RT_5%_), 12% contrast (RT_12%_), 25% contrast (RT_25%_), and 35% contrast (RT_35%_) were analyzed. The test–retest repeatability of EFT was assessed by the intraclass correlation coefficient (ICC). Ten (10 eyes) normal volunteers were tested twice with an interval of 4 weeks.

### Data collection

Demographics and clinical information included age, gender, BCVA, and IOP. MD, mean sensitivity (MS), and loss variance (LV) from automated perimetry and the RNFLT from OCT analysis were collected. BCVA was measured monocularly and was recorded as the logarithm of minimum angular resolution (LogMAR). Only a reliable visual field report was used. A cutoff of 20% for fixation losses and 15% for false-positive response rates were suggested for reliable visual field results [26, 27]. Peripapillary RNFLT was calculated automatically from a single circular B-scan consisting of 1536 A-scans [28, 29]. The RNFLT was reported as the general, inferior, superior, nasal, and temporal RNFLT. The general RNFLT was defined as the average of the RNFLTs of the other four quadrants. All the data were collected within 3 months. Then, the eyes were further classified as early, moderate, advanced, severe, and end-stage glaucoma based on the Enhanced Glaucoma Severity Staging System (eGSS) [30]. Visual field test result is plotted onto a graph with 7 stages in the eGSS according to the main visual field global parameters, such as mean deviation, corrected pattern standard deviation, or loss variance [31]. And the eGSS can be applied to visual field tests from all machines which report these parameters [31].

### Statistical analysis

Statistical analysis was performed using SPSS 25.0 (SPSS Inc., USA), MedCalc software 20.027 (MedCalc Software, Belgium), and R 3.6.1 (The R Foundation, Austria). Data are expressed as the means ± standard deviation. Normality was assessed using the Shapiro–Wilk test. Test–retest repeatability of EFT was assessed by the ICC. Clinical characteristics of the control and different POAG subgroups were compared using the Kruskal–Wallis *H* test. The RT of the control and different POAG subgroups were further compared using the Mann–Whitney *U* test. Kendall’s tau-b correlation analysis was used to analyze the correlation between the different stages of POAG and RT with different test contrasts. The generalized estimating equation was performed to evaluate the relationships between RT and other parameters. Receiver operator characteristic (ROC) curves were calculated from the sensitivity and specificity, and the area under the curve (AUC) values were calculated to estimate the accuracy of glaucoma diagnosis with respect to the subset of nonglaucoma controls. Statistical comparison of AUC of ROC curves were performed by DeLong’s test. Multivariate logistic regression analysis was performed to select significant risk factors for end-stage glaucoma-related indicators. A nomogram model was developed to assigns corresponding scores to each predictor based on regression coefficients. The total score is obtained by adding all the index scores, and the maximum value is 100 points. The accuracy of the model was tested by the consistency index (C-index) and the calibration chart for the bootstrap samples.

## Results

A total of 45 POAG patients (80 eyes) were enrolled in this study, including 25 males and 20 females, with an average age of 61.7 ± 14.6 years (25–84 years). Twenty normal controls (20 eyes), including 8 males and 12 females, with an average age of 43.7 ± 19.4 years old (23–79 years old) were enrolled in this study. Demographics and clinical characteristics are displayed in Table 1. There were no significant differences in gender or IOP between the POAG group and the control group (*P* > 0.05), but there were significant differences in age, visual acuity, MS, MD, LV, and mean RNFL thickness (*P* < 0.05).

**Table 1:** Demographics and clinical characteristics

	POAG group (*n* = 80)	Control group (*n* = 20)	U/χ2	*P*
Age (years)	61.7 ± 14.6	43.7 ± 19.4	211.5	0.001^a^
Gender (male/female)	25/20	8/12	1.340	0.187^b^
BCVA (LogMAR)	0.313 ± 0.336	0.129 ± 0.206	452.5	0.002^a^
IOP (mmHg)	14.78 ± 3.11	15.2 ± 2.68	670.0	0.259^a^
MS (dB)	12.5 ± 7.51	24.8 ± 2.67	72.0	< 0.001^a^
MD (dB)	15.0 ± 7.38	3.49 ± 2.03	84.5	< 0.001^a^
LV	35.5 ± 25.8	6.17 ± 3.95	143.0	< 0.001^a^
Mean RNFLT (μm)	65.2 ± 22.8	97.0 ± 6.83	70.5	< 0.001^a^

^a^Statistical analysis performed by the Mann–Whitney *U* test

^b^Statistical analysis performed by the chi-square test; *BCVA* best corrected visual acuity, *IOP* intraocular pressure, *MD* mean deviation, *MS* mean sensitivity, *LV* loss variance, *RNFLT* retinal nerve fiber layer thickness

### Test–retest repeatability of the EFT

The reproducibility of EFT was good with the test contrast of 3%, 5%, and 12%. And the reproducibility was moderate for the EFT with the test contrast of 25% and 35%. For the control group, the ICC were 0.819, 0.765, 0.767, 0.660, and 0.643 for the EFT with a test contrast of 3% (*P* < 0.001), 5% (*P* = 0.003), 12% (*P* = 0.003), 25% (*P* = 0.017), and 35% (*P* = 0.008), respectively.

### RT in the control group

**Fig. 1:**
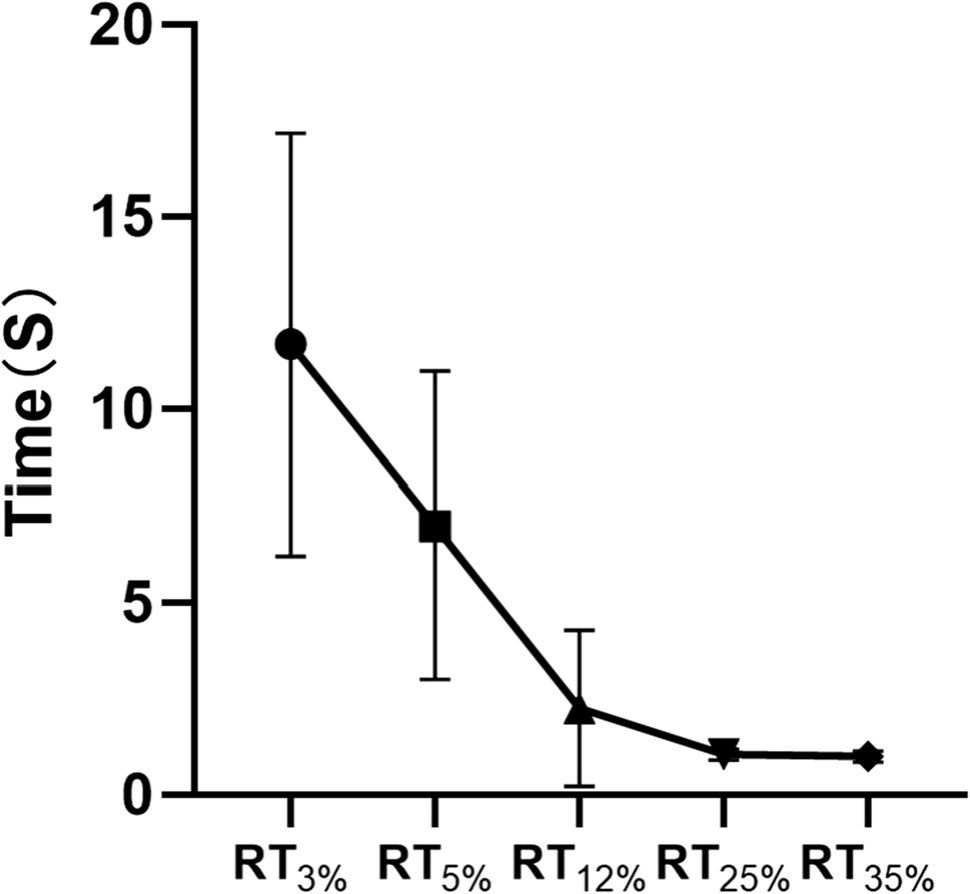
Distribution of RT in different contrasts in the control group

In this study, the average age of the control group was 43.7 ± 19.4 years, ranging from 23 to 79 years. There was no significant correlation between RT_3%_, RT_5%_, RT_12%_, RT_25%_, RT_35%_, and age (rs = 0.175, *P* = 0.462; rs = 0.034, *P* = 0.887; rs = 0.392, *P* = 0.088; rs = 0.372, *P* = 0.107; rs = 0.413, *P* = 0.07). The distribution of the RT in different contrasts in the control group is shown in Fig. 1. As the contrast of the EFT test stimulus increased, the RT gradually decreased.

### Analysis of EFT results in POAG subgroups

POAG eyes were divided into 5 groups according to eGSS (Table. 2). The results of structural and functional tests for glaucoma eyes were compared among different groups. There were significant differences in the BCVA, MS, MD, and LV among the different POAG subgroups. The mean RNFL thickness and the RNFL thickness of each quadrant also showed significant differences in different POAG subgroups (*P* < 0.05).

**Table 2:** Clinical characteristics of POAG subgroups

	Early glaucoma (*n* = 6)	Moderate glaucoma (*n* = 9)	Advanced glaucoma (*n* = 17)	Severe glaucoma (*n* = 24)	End-stage glaucoma (*n* = 24)	*H*	*P*
BCVA (logMAR)	0.136 ± 0.107	0.198 ± 0.153	0.242 ± 0.218	0.266 ± 0.300	0.500 ± 0.444	10.08	0.039
IOP (mmHg)	16.7 ± 3.88	14.3 ± 3.61	14.7 ± 2.97	15.2 ± 3.05	14.1 ± 2.89	5.607	0.230
MS (dB)	24.0 ± 0.488	21.5 ± 1.18	17.8 ± 2.14	11.5 ± 2.28	3.28 ± 2.49	71.831	< 0.001
MD (dB)	3.63 ± 5.75	5.73 ± 1.24	9.87 ± 1.87	15.9 ± 2.28	24.1 ± 2.39	72.548	< 0.001
LV	6.45 ± 3.25	14.1 ± 5.47	36.2 ± 16.6	59.0 ± 23.6	26.1 ± 21.3	41.766	< 0.001
RNFLT(μm)							
Mean RNFLT	93.1 ± 4.55	88.6 ± 17.3	81.7 ± 23.6	53.9 ± 9.89	47.5 ± 10.2	42.954	< 0.001
Inferior RNFLT	109 ± 12.7	107.1 ± 35.5	98.9 ± 34.4	57.9 ± 16.6	52.3 ± 13.9	37.516	< 0.001
Superior RNFLT	114 ± 9.71	116 ± 35.4	104.8 ± 34.8	58.7 ± 19.4	52.8 ± 13.3	40.275	< 0.001
Nasal RNFLT	67.2 ± 10.3	65.3 ± 22.5	55.4 ± 19.3	45.6 ± 14.7	37.3 ± 13.4	28.451	< 0.001
Temporal RNFLT	81.5 ± 16.3	65.4 ± 11.4	57.8 ± 18.0	53.5 ± 23.7	47.7 ± 12.5	21.840	< 0.001

Statistical analysis performed by the Kruskal–Wallis *H* test; *BCVA* best corrected visual acuity, *IOP* intraocular pressure, *MD* mean deviation, *MS* mean sensitivity, *LV* loss variance, *RNFLT* retinal nerve fiber layer thickness

Within 50 s, 1 of the eyes in the advanced glaucoma group could not perceive test stimuli at 12% and 25% contrast. Among the eyes with end-stage glaucoma, 8 eyes could not perceive the test stimulus at 12% contrast, 6 eyes could not perceive the test stimulus at 25% contrast, and 3 eyes could not perceive the test stimulus at 35% contrast. With increasing test contrast, the ability of the POAG eye to perceive the test stimulus in the late and end stages improved.

The Kruskal–Wallis test was used to compare the differences between the POAG subgroups and the control group. There were significant differences between the RT in the severe, end-stage POAG groups, and the control group at 12%, 25%, and 35% test contrast (*P* < 0.001). However, there were no significant differences between RT in the early, moderate, advanced groups, and the control group (*P* > 0.05) (Table. 3). The results were Bonferroni corrected.

**Table 3:** Analysis of EFT results in POAG subgroups

RT (second)	*n*	12%	*n*	25%	*n*	35%
Control	20	2.26 ± 2.03	20	1.05 ± 0.14	20	1.00 ± 0.14
Early glaucoma	6	2.21 ± 1.45	6	1.17 ± 0.53	6	0.82 ± 0.22
Moderate glaucoma	9	8.33 ± 7.39	9	4.39 ± 4.97	9	2.96 ± 3.29
Advanced glaucoma	17	7.38 ± 5.51	17	3.35 ± 2.93	17	1.95 ± 1.88
Severe glaucoma	23	13.0 ± 8.40	23	9.62 ± 6.89	24	6.92 ± 6.27
End-stage glaucoma	16	18.3 ± 7.61	18	14.6 ± 6.63	21	13.1 ± 9.39
*P*_*1*_		1.000		1.000		1.000
*P*_*2*_		0.289		1.000		1.000
*P*_*3*_		0.104		0.419		1.000
*P*_*4*_		< 0.001		< 0.001		0.001
*P*_*5*_		< 0.001		< 0.001		< 0.001

Statistical analysis was performed by the Kruskal–Wallis *H* test, and the results were Bonferroni corrected. *P*_*1*_, control vs. early glaucoma; *P*_*2*_, control vs. moderate glaucoma; *P*_*3*_, control vs. advanced glaucoma; *P*_*4*_, control vs. severe glaucoma; *P*_*5*_, control vs. end-stage glaucoma; *RT* recovery time

**Fig. 2:**
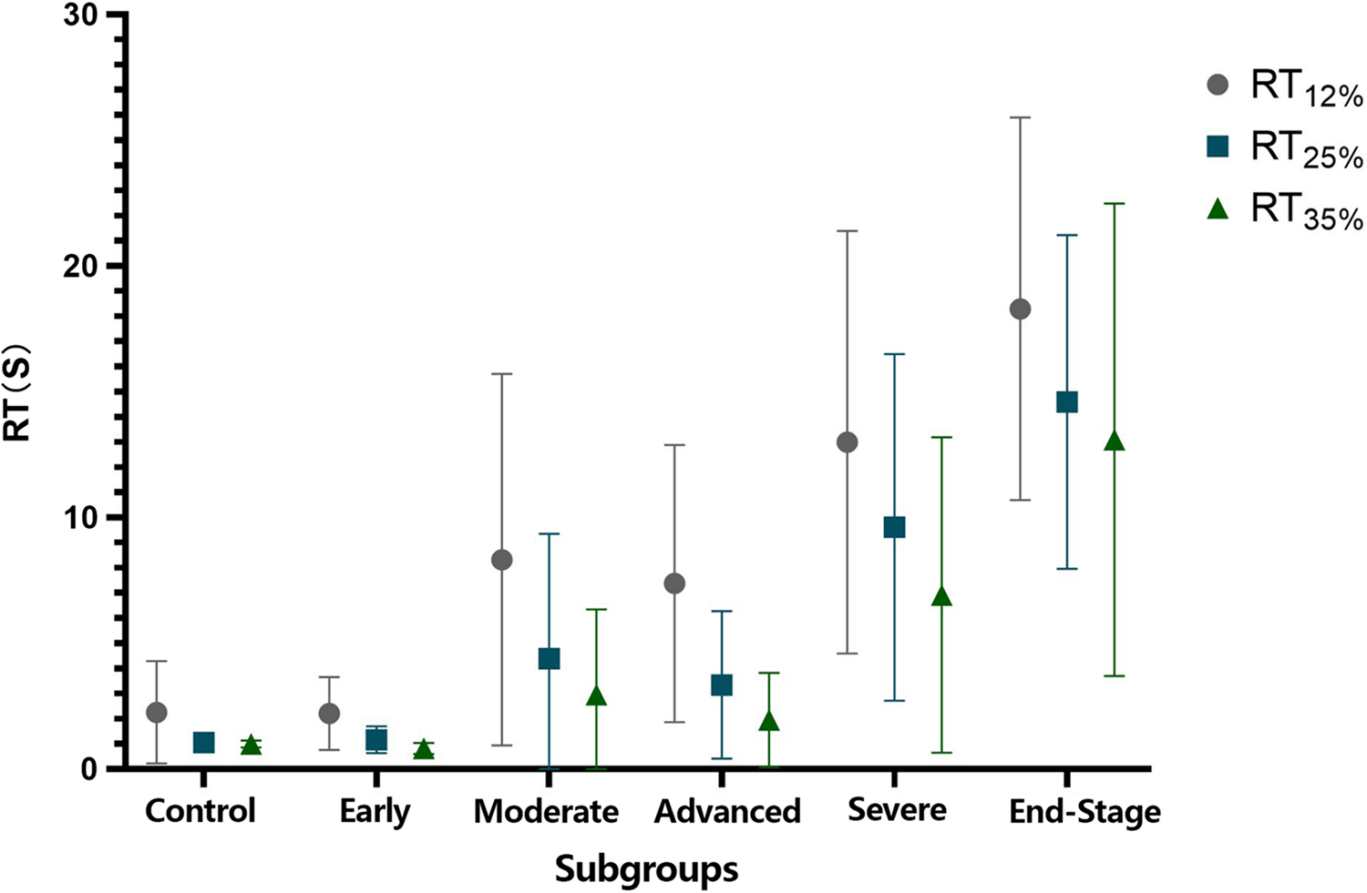
RT in different test contrasts in the control group and different stages of POAG groups

RT in different test contrasts in the control group and different stages of POAG groups were shown in Fig. 2. Kendall’s tau-b correlation analysis was used to analyze the correlation between the different stages of POAG and RT with different test contrasts. RT_12%_, RT_25%_, and RT_35%_ were positively correlated with POAG visual field staging, respectively (Kendall’s tau-b = 0.546, *P* < 0.001; Kendall’s tau-b = 0.591, *P* < 0.001; Kendall’s tau-b = 0.561, *P* < 0.001).

**Fig. 3:**
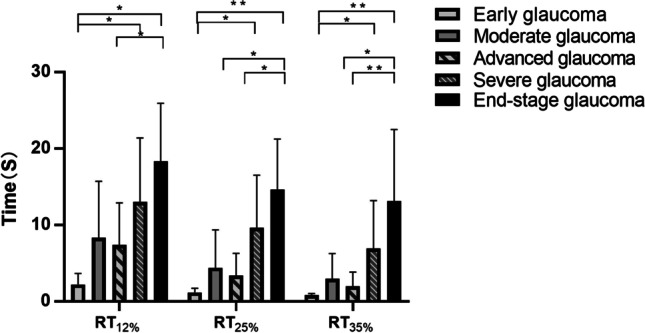
RT_12%_, RT_25%_ and RT_35%_ in different POAG subgroups. **P < 0.001; *P < 0.05

The Kruskal–Wallis test was used to compare the differences in the EFT results among different POAG subgroups (Fig. 3). The results were Bonferroni corrected. At contrasts of 25% and 35%, the RT of end-stage glaucoma was quite different from that of the other POAG subgroups.

### Correlations between RT and structural and functional measurements

Generalized estimating equation was used to analyze the correlations between the RT at different contrasts ​​and glaucomatous structural and functional monitoring indicators (BCVA, IOP, MD, and RNFLT). The results are shown in Table 4. The RT at different contrasts was significantly correlated with BCVA (*P* ≤ 0.001). There was no significant correlation between IOP and RT_12%_, RT_25%_, and RT_35%_ (*P* = 0.721, *P* = 0.973, *P* = 0.189). Among the visual field indicators, MS was significantly correlated with RT_12%_, RT_25%_, and RT_35%_ (*P* < 0.001); MD was significantly correlated with RT_12%_, RT_25%_, and RT_35%_ (*P* < 0.001); and LV was significantly correlated with RT_12%_, RT_25%_, and RT_35%_ (*P* < 0.05). The general thickness of the RNFL and the thickness of the RNFL in each quadrant were significantly correlated with the RT at different contrasts (*P* < 0.05).

**Table 4:** Correlations between RT at different contrasts and glaucomatous structural and functional measures

RT (second)		12%		25%		35%	
		OR	*P*	OR	*P*	OR	*P*
BCVA (LogMAR)		5.067	< 0.001	4.776	< 0.001	7.206	< 0.001
IOP (mmHg)		0.984	0.721	1.002	0.973	0.935	0.189
MS (dB)		0.909	< 0.001	0.892	< 0.001	0.899	< 0.001
MD (dB)		1.102	< 0.001	1.123	< 0.001	1.115	< 0.001
LV		1.023	< 0.001	1.022	< 0.001	1.011	0.009
RNFLT (μm)	General	0.980	< 0.001	0.977	0.001	0.974	< 0.001
	Superior	0.987	< 0.001	0.984	0.001	0.982	< 0.001
	Inferior	0.987	< 0.001	0.986	0.002	0.983	< 0.001
	Nasal	0.979	< 0.001	0.972	< 0.001	0.973	< 0.001
	Temporal	0.986	0.019	0.984	0.025	0.966	0.003

Statistical analysis performed by the Generalized estimating equation; *RT* recovery time, *BCVA* best corrected visual acuity, *IOP* intraocular pressure, *MD* mean deviation, *MS* mean sensitivity, *LV* loss variance, *RNFLT* retinal nerve fiber layer thickness

**Fig. 4:**
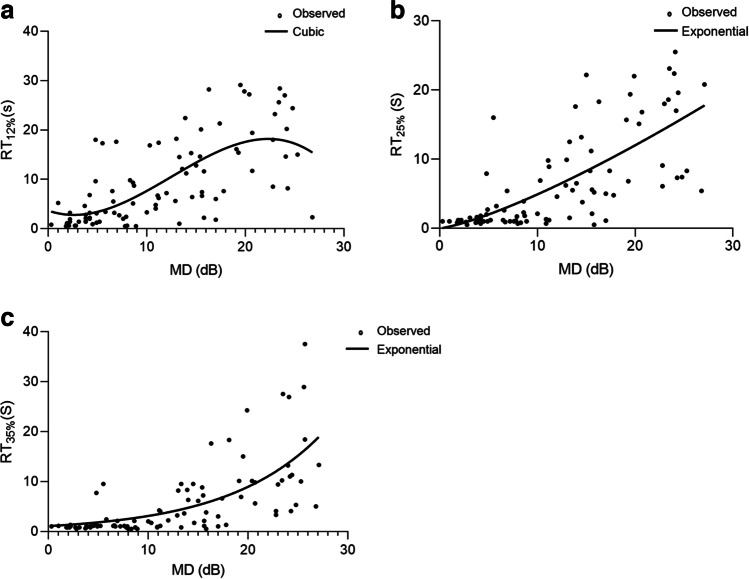
Scatterplot and correlation models between RT and MD. **A** Scatterplot and correlation models between RT_12%_ and MD. **B** Scatterplot and correlation models between RT_25%_ and MD. **C** Scatterplot and correlation models between RT_35%_ and MD

Correlation models between RT and structural and functional measurements were analyzed by regression analysis (Table. 5). The scatterplots of RT_12%_ and MD were best fitted by a cubic model with a higher *R*^2^ (Fig. 4A):

**Table 5:** Correlation models between RT and structural and functional measurements

	RT	Cubic		Exponential		Power	
		*R*^2^	*P*	*R*^2^	*P*	*R*^2^	*P*
MD (dB)	12%	0.478	< 0.001	0.448	< 0.001	0.453	< 0.001
	25%	0.515	< 0.001	0.568	< 0.001	0.485	< 0.001
	35%	0.424	< 0.001	0.539	< 0.001	0.416	< 0.001
General RNFLT (μm)	12%	0.327	< 0.001	0.313	< 0.001	0.315	< 0.001
	25%	0.330	< 0.001	0.374	< 0.001	0.370	< 0.001
	35%	0.227	0.003	0.343	< 0.001	0.341	< 0.001
Inferior RNFLT (μm)	12%	0.390	< 0.001	0.354	< 0.001	0.386	0.001
	25%	0.296	< 0.001	0.384	< 0.001	0.410	< 0.001
	35%	0.247	< 0.001	0.361	< 0.001	0.399	< 0.001
Superior RNFLT (μm)	12%	0.299	< 0.001	0.193	< 0.001	0.210	< 0.001
	25%	0.285	< 0.001	0.257	< 0.001	0.260	< 0.001
	35%	0.207	0.001	0.256	< 0.001	0.256	< 0.001
Nasal RNFLT (μm)	12%	0.218	< 0.001	0.158	< 0.001	0.106	0.004
	25%	0.244	< 0.001	0.210	< 0.001	0.137	< 0.001
	35%	0.151	0.007	0.162	< 0.001	0.109	0.003
Temporal RNFLT (μm)	12%	0.295	< 0.001	0.169	< 0.001	0.107	0.004
	25%	0.240	< 0.001	0.184	< 0.001	0.116	0.002
	35%	0.122	0.023	0.159	< 0.001	0.100	0.005

Statistical analysis was performed by cubic, exponential, and power regression analyses; *RT* recovery time, *BCVA* best corrected visual acuity, *IOP* intraocular pressure, *MD* mean deviation, *MS* mean sensitivity, *LV* loss variance, *RNFLT* retinal nerve fiber layer thickness

$$y=-{0.004x}^{3}+{0.150x}^{2}-0.706x+3.679$$

The scatterplots of RT_25%_ and MD were best fitted by an exponential model with a higher *R*^2^ (Fig. 4B):

$$y=0.824\times {e}^{0.121x}$$

The relationship between RT_35%_ and MD was well explained by an exponential model with a higher *R*^2^ (Fig. 4C):

$$y=0.668\times {e}^{0.110x}$$

### ROC analysis

**Fig. 5:**
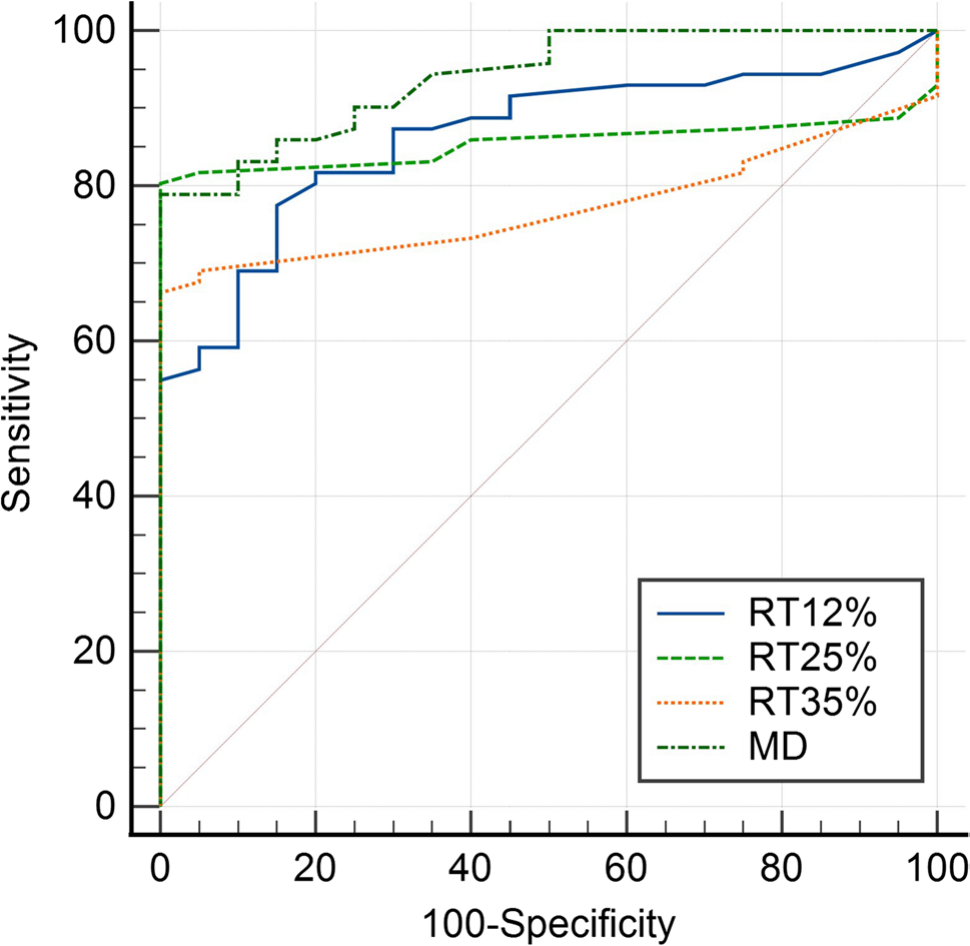
ROC curves for RT_12%_, RT_25%_, RT_35%_, and MD

ROC analysis was performed for RT_12%_, RT_25%_, RT_35%_, and MD. Among the RTs, RT_12%_ showed the best performance for diagnosing POAG, with an AUC of 0.863, followed by RT_25%_ with an AUC of 0.855. RT_35%_ had the worst diagnostic value with an AUC of 0.772. And the AUC for MD was 0.940 (Fig. 5). DeLong’s test for ROC curves demonstrated significant differences between RT_12%_ and RT_35%_ (Z = 2.409, *P* = 0.016) and RT_25%_ and RT_35%_ (Z = 2.595, *P* = 0.0095). And there was no significant difference in the ROC curve between RT_12%_ and RT_25%_ (Z = 0.188, *P* = 0.85).

### POAG staging based on EFT and OCT

**Fig. 6:**
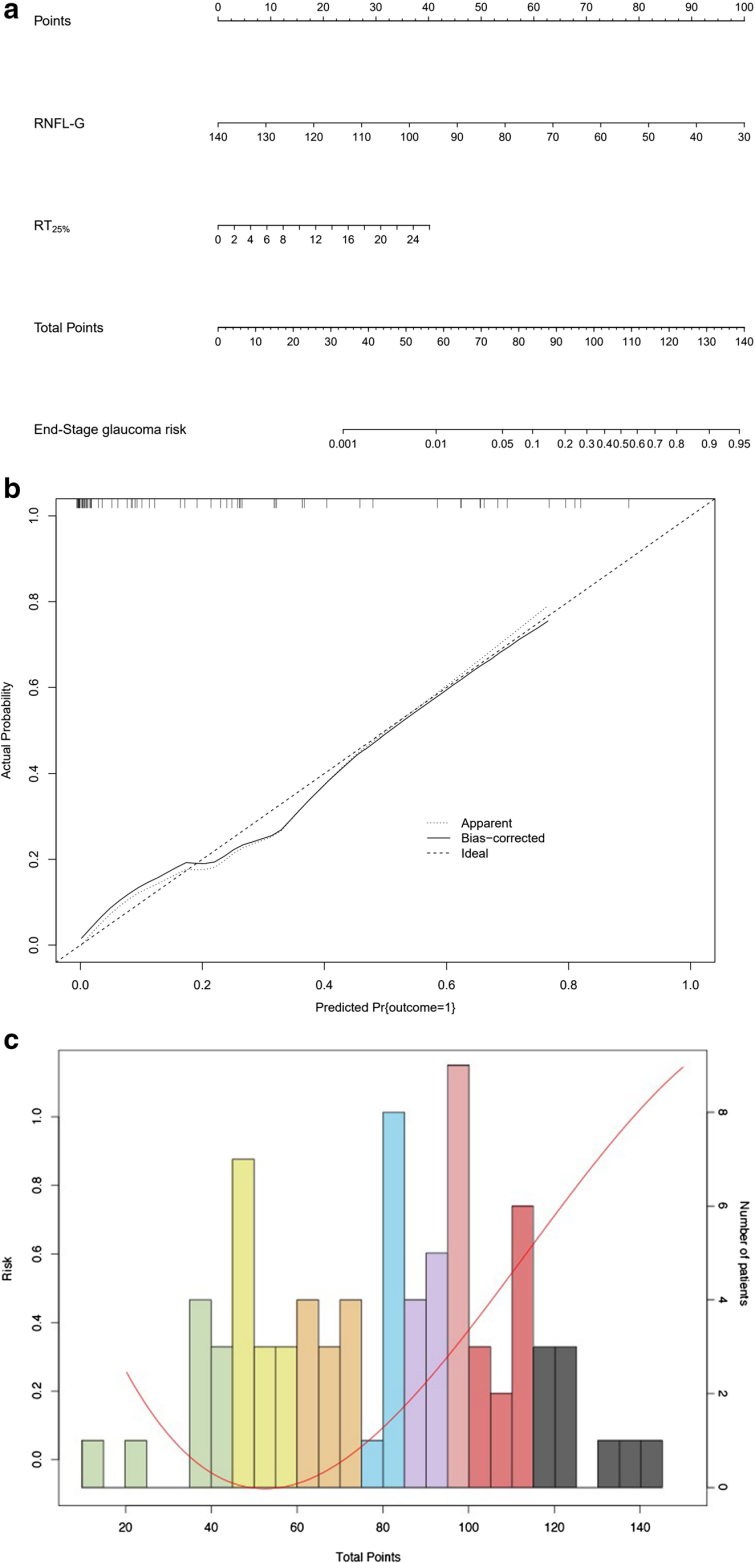
**A** For predicting the probability of end-stage glaucoma, RT_25%_ and average RNFL thickness were included and placed on the variable axes. Draw vertical lines of each risk factor towards the total point scale, which was assigned to be the point as each parameter. Then sum up the points of the parameters as total points. **B** The calibration plot showed that the model curve fit well with the standard curve. **C** Risk curve based on the total points refers to the probability of end-stage glaucoma

POAG eyes were further divided into two groups according to the eGSS: the first group included early, moderate, advanced, and severe glaucoma eyes, and the second group included end-stage glaucoma eyes. The characteristics of end-stage glaucoma were analyzed. Compared to the first group, the RT_25%_ of the end-stage glaucoma was significantly increased, and the general RNFL thickness was significantly reduced (*P* < 0.05) (Table. 6). Therefore, this study used RT_25%_ and average RNFL thickness to develop a nomogram model. These two indicators correspond to the scores. A probability axis was drawn based on the relationship between total points and the probability of end-stage glaucoma (Fig. 6A). The nomogram demonstrated relatively good performance, with a C index of 0.77, and the calibration plot showed that the model curve fit well with the standard curve (Fig. 6B). According to the nomogram, the POAG eyes could be further divided into 8 stages (Fig. 6C): Patients with or fewer than 45 points were defined as stage 1, patients with 45–60 points or with 60 points were defined as stage 2, patients with 60–75 points or with 75 points were defined as stage 3, patients with 75–85 points or with 85 points were defined as stage 4, patients with 85–95 points or with 95 points were defined as stage 5, patients with 95–100 points or with 100 points were defined as stage 6, patients with 100–115 points or with 115 points were defined as stage 7, and patients with more than 115 points were defined as stage 8. According to the probability distribution (the red line), this model was less effective in evaluating the visual function of early glaucoma. As the disease progressed, it became more accurate.

**Table 6:** The multivariate logistic regression analysis of end-stage glaucoma

	Odds ratio (95% CI)	*P*
RT_25%_	1.156 (1.041–1.283)	0.007
General RNFLT	0.918 (0.854–0.985)	0.018

Statistical analysis performed by multivariate logistic regression analysis. *RT* recovery time, *RNFLT* retinal nerve fiber layer thickness

## Discussion

The current study used EFT to explore temporal contrast sensitivity in POAG eyes, especially in advanced and end-stage disease. We found that after 25 Hz adaptation, the EFT had poor performance in classifying early and moderate glaucoma but better performance in evaluating visual function for advanced and end-stage glaucoma.

It is important for a glaucomatous screening test to be capable of differentiating diseased from normal individuals [32]. In the current study, test–retest reliability was assessed using the ICC. The EFT showed good reproducibility with the test contrast of 3%, 5%, and 12% and showed moderate reproducibility with the test contrast of 25% and 35%, which were acceptable for a clinical test. During the test process, we strengthened the guidance and supervision of patients to ensure the accuracy of the test results. Several studies have attempted to explore temporal contrast sensitivity in normal eyes. Horn et al. [33] found that in the control group, there was no significant age relationship with the temporal contrast threshold. However, Brehmer et al. [23] showed that both RT_3%_ and RT_5%_ were significantly correlated with age. Cursiefen et al. [34] found that although the RT increased 0.55 s per decade, there was no significant correlation between RT and age. The current study also found that there was no significant correlation between RT and age in normal eyes. Compared to previous studies, the current study involved more test contrast. Subsequent studies should include more controls and further investigate the correlation between RT and age. The current study also found that, as the contrast gradually increased, the RT showed a downward trend. This might be related to the temporal contrast adaptation and stimulation threshold of M-cells [21, 22, 35]; that is, for 100% contrast adaptation stimuli at 25 Hz frequency, magnocellular cells protect themselves by preventing overstimulation, while lower contrast test stimuli might not be able to be perceived by damaged RGCs. Brehmer et al.[23] found that RT_3%_ and RT_5%_ in the early glaucoma group (mean MD = 6.33 dB) were significantly prolonged compared with the control group, but there was no significant difference between the preperimetric POAG group and the control group. The study also found that in the control group, RT_3%_ was greater than 10 s and RT_5%_ was greater than 8 s, while in the early glaucoma group, RT_3%_ was greater than 20 s and RT_5%_ was greater than 10 s. It showed that RT_3%_ and RT_5%_ could distinguish glaucoma patients from normal people and could be used as auxiliary indicators for glaucoma diagnosis. However, under low contrast (3%, 5%), glaucoma patients needed a longer time to perceived the contrast test stimulus. It may take 30 min to complete an accurate test under the low contrast for glaucoma patients. Considering the limited time and resources of outpatient examinations in our country, EFT under low contrast was not suitable for outpatient. These were the reasons why RT_3%_ and RT_5%_ were not obtained for glaucoma eyes in the current study.

In the current study, POAG eyes were classified into 5 groups according to the eGSS. Brehmer et al.[23] found that the RT_12%_, RT_25%_, and RT_35%_ of advanced glaucoma (mean MD = 19.26 dB) eyes were significantly prolonged compared with normal eyes. The current study included more advanced and end-stage POAG eyes than Brehmer et al. [23], with an average MD of 14.9 dB. We found that RT_12%_, RT_25%_, and RT_35%_ of the early, moderate, and advanced POAG were not significantly different from those in the control group, suggesting that EFT with 12%, 25%, and 35% test stimuli had poor diagnostic efficiency for these stages of glaucoma. As the disease progressed, RT_12%_, RT_25%_, and RT_35%_ could better distinguish POAG eyes from control eyes, and the RTs under the three contrasts were significantly different from those of the control group. Therefore, under contrasts of 12%, 25%, and 35%, the EFT had better diagnostic efficiency for advanced and end-stage glaucoma. Based on a comparison of the AUC values of the EFT results, RT_12%_ had better diagnostic efficacy for POAG, followed by RT_25%_ and RT_35%_. Brehmer et al. [23] reported that the AUC of RT_3%_ was 0.734 and the AUC of RT_5%_ was 0.684. However, in the current study, the ROC analysis included all POAG patients in the diagnostic efficacy analysis, which might overpredict the diagnostic efficacy of EFT. In the current study, POAG was diagnosed according to the guidelines of the American Academy of Ophthalmology [24], including the visual field results on SAP, so the parameter from SAP showed higher AUC, and we included it as the standard of reference. Therefore, took the results of previous study into consideration; we propose that the RT under low contrast (3% and 5%) might be useful in assisting the clinical diagnosis of early and moderate glaucoma, while the RT under higher contrast (12%, 25%, and 35%) might be an alternative option for the clinical diagnosis of advanced, severe, and end-stage glaucoma.

Furthermore, RT at different contrasts increased gradually as the severity of glaucoma disease progressed. The results of the current study suggested that the closer to the end stage, the more significant the difference in RT, especially RT under 25% and 35% contrast, which could better distinguish severe and end-stage glaucoma from other stages, indicating that RT_25%_ and RT_35%_ could be used as POAG staging indicators, especially for the classification of severe and end-stage glaucoma. Therefore, the EFT might be useful for the visual function monitoring in severe and end-stage glaucoma and might be helpful in the adjustment of clinical treatment strategies, which could prevent further visual function damage.

Another finding of the current study was the associations among the results of the glaucoma functional tests such as the BCVA and SAP and of the structure tests such as OCT. In the current study, the RT at all three different contrasts was negatively correlated with MS and RNFL and was positively correlated with MD and BCVA. SAP is widely accepted in clinical practice, as it can provide reasonably reliable disease staging and monitoring for glaucoma [36]. The overall performance of the RT from the EFT was compared with that of MD from SAP. According to the scatterplots with model fits of the relationships between MD and RT, when MD was greater than 22.4 dB, RT_12%_ began to decrease, indicating that when the visual field defect reached a certain degree, RT_12%_ could not continue to monitor the visual function damage. Therefore, RT_12%_ cannot be used to monitor visual function damage in end-stage glaucoma. However, RT_25%_ and RT_35%_ continued to increase as the MD increased. Therefore, RT_25%_ and RT_35%_ could be used in visual function monitoring in all stages of POAG.

Particularly, if the result from SAP is not reliable, especially for those patients who are unable to complete the test or have poor fixation, the EFT could serve as a supplementary functional test. Regarding the structural parameters, the RT at the three different contrasts yielded significant associations with the RNFLT in different quadrants. These findings revealed that the RT was related to the severity of glaucomatous visual function impairment. Therefore, for patients with disease progression, the RT, especially RT_25%_ and RT_35%_, could be used as a supplement to structural and functional tests for disease monitoring and staging.

At present, clinical monitoring for glaucomatous damage in patients with end-stage glaucoma depends on the doctor’s experience or the patient’s visual acuity [4]. However, for some patients with a tubular visual field or only a small part of the visual field, it is more difficult to detect disease progression [37]. Therefore, the current study included visual function and structural indicators to construct a nomogram for visual function and structural assessment in end-stage POAG patients. After regression analysis, we developed a nomogram using RT_25%_ and average RNFL thickness. In end-stage glaucoma, RNFL thickness may reach a “floor effect” and cannot be used independently as an indicator in disease monitoring. RT_25%_ was used as an indicator to evaluate visual function in severe and end-stage glaucoma. Using the nomogram to estimate glaucomatous damage is a new concept. Combined with glaucoma structural evaluation indicators, the risk score in the nomogram can help doctors assess glaucomatous damage and classify end-stage glaucoma further. Through the nomogram, it can be found that in eyes with early glaucoma, as the total score increases, the risk of visual function and structural damage decreases. This model further divides the moderate- and end-stage POAG into 6 stages. For some patients with higher scores in the end-stage, the visual function and structure can be evaluated according to the risk score, which may be helpful in adjusting the treatment strategy in time and preventing irreversible blindness.

There were several limitations to this study. Only POAG patients were included, and different types of glaucoma, such as angle-closure glaucoma or secondary glaucoma, should be included to further explore the diagnostic value of the EFT. Due to the limited follow-up period, this study is a cross-sectional study. Long-term follow-up was not performed to observe disease progression. Therefore, the efficacy of EFT in disease progression monitoring cannot be evaluated. In addition, the sample size was relatively small. Prospective clinical trials with larger sample sizes are still needed for further confirmation. Although the EFT has advantages such as a short duration, it is subjective. Objective examinations are still needed for disease monitoring to achieve higher accuracy.

## Conclusions

In summary, as an electrophysiological examination, combined with traditional glaucomatous structural and functional parameters, the EFT can be used in the diagnosis and visual function analysis of POAG, especially for severe and end-stage glaucoma. It could be used as a potential test for disease staging in severe and end-stage glaucoma, which might further guide clinical treatment and prevent irreversible blindness.

## Data Availability

The datasets used and analyzed during the current study are available from the corresponding author on reasonable request.
